# Applying and assessing the PEERS model on genetic counseling training in China: A mixed-method approach

**DOI:** 10.3389/fmed.2022.986851

**Published:** 2023-01-11

**Authors:** Dong Dong, Shiyi Xiong, Elena Nichini, Xiaoqiong Chen, Binjuan Liu, Liling Zhu, Faustina Fu Yip, Luming Sun, Jianfeng Zhu

**Affiliations:** ^1^JC School of Public Health and Primary Care, Faculty of Medicine, The Chinese University of Hong Kong, Shatin, Hong Kong SAR, China; ^2^Shenzhen Research Institute, The Chinese University of Hong Kong, Shenzhen, Guangdong, China; ^3^Shanghai Key Laboratory of Maternal Fetal Medicine, Department of Fetal Medicine and Prenatal Diagnosis Center, Shanghai First Maternity and Infant Hospital, School of Medicine, Tongji University, Shanghai, China; ^4^Fudan-Harvard Medical Anthropology Collaborative Research Center, School of Social Development and Public Policy, Fudan University, Shanghai, China; ^5^Department of Population Health Sciences, Bristol Medical School, University of Bristol, Bristol, United Kingdom

**Keywords:** genetic counseling, training, China, mixed-method approach, counselor–patient communication, PEERS model

## Abstract

**Objective:**

Due to the changing medical demands in the healthcare system, there is a need for a standardized and professionalized curriculum for genetic counselors. This mixed-method study will observe and evaluate the first Peer Experiential and Reciprocal Supervision (PEERS) training program on genetic counseling among medical practitioners in China; to provide feedback and recommendation for future training and practices.

**Methods:**

A genetic counselor training program was held from December 10–11, 2016 in a fetal medicine unit and prenatal diagnosis center in Shanghai with 59 participants from clinical centers, hospitals, and organizations in China. An ethnographic reflexive assessment with a structured questionnaire were used to provide insights and feedback on the training experience.

**Results:**

Results indicate an inadequate mastery of genetic and fetal knowledge; lack of empathetic understanding and cultural sensitivity; difficulties in adopting a non-directive counseling approach; distance between reality and fictionality in the training; overall training's helpfulness.

**Conclusion:**

The professionalization of genetic counseling in China is in the making with the soaring demands for genetic counseling services; this first experiment of PEERS training turned out to be needed, worth to be adapted toward medical centers across China, to better understand and face the challenges rising from genetic counseling practice.

## Introduction

Genetic counseling is a process that helps people understand and cope with medical, psychological, and family consequences attributable to genetic diseases. Conceived in the United States in 1969, genetic counseling has now been institutionalized as a profession in no less than 28 countries with 7,000 certified professionals worldwide (1). Yet China is not one of them; due to the disconnection between genetic research and application in both clinical and educational settings, genetic counseling has in fact still not been professionalized (2). There is neither a state-sanctioned certified genetic counseling training program at the college level, nor is there a more specialized training curriculum focusing on counselor–patient communication. Although there has been a growing demand in the Chinese medical system for genetic counselors, it was not until 2020 that Fudan University in Shanghai initiated China's first postgraduate genetic counseling program (3). However, this master program is only offered to part-time students. Even as the Chinese government had set up guidelines to regulate genetic counseling in 2003, the practice and application of it is done by a clinical geneticist who are, trained obstetricians and pediatricians (4). These clinicians are medical providers with specific training in clinical genetics who would be able to diagnose and analyze test results and estimate risks “Quasi genetic counselors,” who only provided services on traditional genetic diseases (e.g., Down Syndrome) by regular genetic testing (e.g., karyotype analysis), are now providing services to meet the growing demands from the patients who need to understand the genetic issues that they have encountered (2). Most of these quasi counselors work in big cities such as Shanghai, Beijing, or Guangzhou, and most are clinicians without a systematic education in clinical genetics (1). To meet this growing resource gap in the clinical setting, many healthcare professionals in China, such as OBGYNs and pediatricians have looked into and taken up certificate courses from gene technology companies (e.g., BGI, Berry Genomics) with or without in collaboration with medical associations instead (5–8).

However, the year 2015 has been a turning point in many aspects. First, with the announcement of a full implementation of the “two child policy” in China on October 29 (which was later changed into a “three child policy” in May 2021), it was expected that the increasing number of newborns would result in a rising rate of birth defects and genetic disorders due to advanced parental age (2). Meanwhile, the National Health Commission issued permission to 109 institutes to conduct pilot non-invasive prenatal testing (NIPT) programs in clinics, and the Chinese Board of Genetic Counseling (CBGC) initiated genetic counseling training courses first in Shanghai and then in several other provinces (2). As a result, the professionalization of genetic counseling is in the making as there is a soaring demand for genetic counseling services.

Yet genetic counseling remains a complex practice, which is often met with challenges and barriers. For instance, through observations and interviews with 156 Mexican pregnant women, Browner et al. identified five sources of miscommunication during prenatal genetic counseling: (1) medical terminology; (2) non-directive nature of counseling; (3) genetic counselors' over-sensitivity to patients' culture; (4) translation issues; and (5) trust issues (9). According to the National Society of Genetic Counselors (NSGC), genetic counseling should respect the autonomy, individuality, wellbeing, and freedom (1) of individuals and their families, but actual counseling sessions are still possibly infused with dilemmas and challenges (10).

For this reason, supervision is a critical part of professional preparation for genetic counseling students, development of clinical counseling technology, and guarantee of standardized care for patients (11, 12); research has then suggested more supervision and training opportunities for both students and practitioners should be developed (13).

According to a survey of clinical supervision in genetic counseling among 348 genetic counselors registered with NSGC in 2000, the most prevalent model in clinical supervision is one-on-one oral feedback. About 82% of the respondents indicated that they always or often provide immediate feedback to their supervisees after a counseling session. Immediate feedback usually focuses on intuitive responses, correction of technical skills (e.g., use of terminology), and micro techniques of consultation (e.g., use of silence). Feedback after a certain period (e.g., 3–4 days later) instead allows the supervisor and the supervisee to be more reflective about the genetic counseling sessions and to focus more on skills at the macro level, such as the counselor's reassurance of patient's emotions and sensitivity to cultural issues (12).

However, one-on-one supervision is only one of the possible models of genetic counseling supervision, which include peer group supervision, leader-led peer group supervision, team supervision (a combined mode of personal supervision and peer supervision), and on-site supervision (14–16). Among all these models, peer group supervision promotes professional and personal development by providing peer support and validation, deepening one's understanding of patients and increasing knowledge and skills, offering opportunities for informal socializing, and fostering a sense of community (17, 18). This study will therefore use a peer group supervision to conduct the feasibility of such an implementation into the training and educational curriculum of its genetic counselors in China.

There are two main approaches to conduct peer group supervision: (1) Peer Observed Interaction and Structured Evaluation (POISE) (19) and (2) Peer Experiential and Reciprocal Supervision for Genetic Counselors (PEERS) (14). Although both POISE and PEERS focus on real-time observation and instant feedback, PEERS model values more on a collaborative, reciprocal relationship by balancing the power relationship between the observer and the consultant. The characteristics of PEERS are as follows: (1) a mutually beneficial model promoted by consultants and observers rather than a more hierarchical relationship as in POISE; (2) learning and practice are achieved through self-awareness and self-reflection rather than relying on a series of formal evaluation materials as in POISE; (3) there is a detailed process for establishing the PEERS model, emphasizing the working relationship of projects among peers; and (4) the observer is present in the room to enhance experiential learning, whereas in the POISE model, observers watch from outside the counseling room through a one-way glass.

The most unique element about the PEERS model is the presence of a third person in addition to the supervisor and the supervisee—an observer—in the room, which will prevent the supervisor or the supervisee from being biased by their own recall and interpretation. Trainees can also further reflect on themselves through the immediate feedback from the observer. Since the observer, the supervisor, and the supervisee share an equal perspective, their exchange of views can more easily promote their own learning and the acceptance of each other's opinions (14). In short, the PEERS model requires the observer and the observed to give feedback to each other and to learn new methods and skills from each other. It can therefore strengthen the supervising skills of the supervisor while facilitating the supervisee to exercise counseling methods and skills at the same time (14).

## 2. Methods

### 2.1. Aims and objectives

The aims of the study were to apply the PEERS model to genetic counseling training programs in China and to evaluate the helpfulness of such a model for medical training in China. The objectives are: (1) to observe and evaluate the application of the PEERS model among genetic counselors in China; (2) to bring ethnographic perspective and observation into its practice and provide an etic view of their performances for both supervisors and the supervisees; and (3) to provide feedback and recommendations for the future development of genetic counseling training and genetic counseling in China more broadly.

### 2.2. Research design

#### 2.2.1. The genetic counselor training course

A genetic counselor training course was held on December 10 and 11, 2016 in Shanghai, China, hosted by the Fetal Medicine Unit and Prenatal diagnosis center, Shanghai First Maternity and Infant Hospital. Fifty-nine participants from a variety of medical institutes across China were enrolled in the course (Supplementary Table 1 on demographics of the trainees). The participants were divided into seven groups, each of which was assigned to one specific scenario on a particular hereditary disease or fetal abnormality to counsel (Table 1). Counseling skills, including lectures or tutorials on medical and genetic aspects were provided before the start of the study. Among the fifty-nine participants, seven of the more experienced physicians were elected and assigned to one group before the training to make sure each group had similar levels of medical and clinical knowledge. The other 52 participants were then randomly assigned to each group. Each group had their own unique scenario in which they were provided with a description 15 min before the simulation. Relevant counseling points were then discussed among each group. Members of each group played three roles: patient, counselor, and observer. For trainees assigned into the same group, they would be roleplaying a counselor and observer where they were to discuss which role they would be assigned to before the simulation. After receiving the script of the counseling scenarios, the “patient,” played by the staff (consisting of seven physicians), was in charge of acting out the role depicted by the scenario and the counselor had to fulfill their duty on counseling in discussing relevant knowledge points with the peer observer required to observe closely to evaluate their members' performance. A feedback session was held after each counseling session where participants could share observations and comments. Further, three medical anthropologists stayed with the same group and observed different members taking rotations in playing the roles.

**Table 1:** Scenarios.

**Scenario 1**	**Singleton pregnant woman, Li Mou, 31 years old, no family history of hereditary diseases, no special medical history, fetal nuchal translucency (NT) is 6.0 mm**.
Scenario 2	Healthy couples, monochorionic diamniotic twin pregnancy, 21-week gestational age, fetal ultrasound resulted in one fetus affected with tetralogy of Fallot.
Scenario 3	Dichorionic diamniotic twin pregnancy, one fetus was diagnosed as Wolf Hirschhorn syndrome by amnio fluid chromosomal test. The other fetus had normal chromosome.
Scenario 4	Pregnant woman, Li Mou, 31 years old, 0-0-1-0, no family history of hereditary diseases, no special medical history, twin pregnancy (chorionicity unknown), F1 fetus NT 6.0 mm, F2 fetus NT 1.3 mm.
Scenario 5	Singleton pregnancy, fetus was diagnosed as Klinefelter syndrome.
Scenario 6	Singleton pregnancy, fetus was diagnosed as Wolf-Hirschhorn syndrome.
Scenario 7	Singleton pregnancy, mosaicism result of 45,X[25]/46,XX[25] found in amniotic chromosome test.

#### 2.2.2. Qualitative evaluation with observations and interviews

A research team constituted of medical anthropologists joined the training, observing the implementation of the PEERS model. Mainly through unobtrusive observations and semi-structured interviews, a qualitative evaluation was conducted on scene. Due to the limitation of time and resources, the team of medical anthropologists fully observed three counseling sessions and interviewed each member of the sessions for half an hour. The entire observation processes and interviews were recorded, transcribed in verbatim and coded. Thematic analysis was employed to analyze and interpret the data (20).

#### 2.2.3. Quantitative evaluation with a structured questionnaire

To assess the training impact and overall experience, participants were asked to fill a structured questionnaire composed of 28 questions, most of which were using a 5-point Likert scale (see supplement for the questionnaire). Questions on the helpfulness of the training sessions included: (1) whether participants learn new skills out of the PEERS training; (2) whether they realized they need to put more efforts in certain practices/skills and their level of competence for specific skills; (3) whether the training led to a better awareness of their counseling style; and (4) their overall satisfaction over the learning experience and suggestions for future training.

## 3. Results

### 3.1. Qualitative evaluation

Fifty-nine participants from a variety of medical institutes were enrolled in the course and divided into seven groups for the PEERS training. Four main themes centering on the challenges for the genetic counselors emerged from the observation and interview data generated by the qualitative research team during the training, namely: (1) inadequate mastery of genetic knowledge; (2) a lack of empathetic understanding and cultural sensitivity; (3) difficulties in providing information in a non-directive manner; and (4) distance between reality and fictionality.

#### 3.1.1. Theme 1: Inadequate mastery of genetic knowledge

The first challenge emerged from the sessions was related to the counselors' mastery of genetic knowledge; insufficient knowledge was reported as an obstacle for effective counseling. For example, for the group training on prenatal diagnosed Klinefelter syndrome (47 XXY), the counselor was not familiar with this chromosomal abnormality. While in this case the doctor would be considered appropriate to refer the patient to a special “genetic counseling clinic” for consultation, from the patient's perspective, the counseling session was not helpful and perceived as poor care since she “needed to make appointments with other clinics and that was really troublesome.”

#### 3.1.2. Theme 2: A lack of empathetic understanding and cultural sensitivity

Lack of empathy and cultural sensitivity for the patients was regarded as the most general issue with consulting skills. For example, it clearly emerged when dealing with religious individuals. In a case of Wolf-Hirschhorn syndrome, the patient was a Muslim woman. The doctor thought that the woman would not terminate pregnancy any way:

*Relatively speaking, her religious belief (Islam) had eased my stress…I thought I only needed to tell her some general things and give her psychological comforts…That should be good enough for the patients*.

As a result, rather than offering more detailed information about the genetic abnormalities of the condition, he only provided the diagnosis and comforted her. Yet such attitude was not embraced positively by the woman:

*The doctor just listened to my personal narration without mentioning any of my religious belief. Because my personal religious belief does not allow me to easily give up this life, I hope he can communicate with me in this regard*.

From the patients' perspective, information is not enough, but should be coupled with personalized guidance to deal with future challenges.

#### 3.1.3. Theme 3: Difficulties in providing information in a non-directive manner

The principle of “non-directiveness” in genetic counseling was regarded as particularly challenging. During the PEERS sessions, participants were in fact often caught in a dilemma. While in the role of patients, guidance was perceived as crucial for decision-making, yet in the role of medical professionals, reluctance to provide any directions emerged:

*The doctor certainly will not give you a directive answer, she can only give you a suggestion. It should be totally up to the patients themselves in terms of dealing with the test results*.

A different conflict emerged when applying a non-directives approach in actual medical settings. Some participants mentioned that the lack of a well-established welfare system in Chinese society might bring burden to families with children with disabilities; for this reason, health professionals would rather openly provide directive recommendations. For example, one participant pointed out that:

*The ideal consultation should be very objective, no emotions, and no suggestions. But in reality, it is not the case. We don't have enough social support for children with Down Syndrome or other disabilities. Our suggestions will affect these families' future. Therefore, sometimes, during the clinical counseling sessions, we would give obviously directive suggestions, telling the patients directedly, such as “Why would you like to rear such a child. Better not to do so.”*

#### 3.1.4. Theme 4: Distance between reality and fictionality

Participants felt “unreal” arrangements during the training may have caused challenges not actually relevant in everyday clinical settings. For instance, while in the training, participants felt pressure on providing immediate answers or suggestions. In actual practice when coming across unfamiliar cases or symptoms, the clinicians would persuade the patient to make another appointment at a later time, which allows them to search medical literature afterwards. Another issue concerns the different attitudes of patients in the training compared to real-time scenarios; for instance, neither trust nor empathy could be easily achieved in such a pseudo setting. Lastly, in real life, counselors were more constrained by time and felt it hard to apply the principles and skills learnt from the training sessions toward real life.

#### 3.1.5. Overall training helpfulness

The qualitative interviews with the participants revealed that they considered the PEERS model a novel way of learning and they had benefited a lot from the training sessions. Even though they felt stressed when being observed by their peers, the feedback they received made it worthwhile. However, such a format made some participants uncomfortable. They suggested that a less stressful way of applying the PEERS model would be to videotape the process and hand it to the participants afterwards, rather than letting them be observed by their peers in real-time:

(*I understand that) there were colleagues observing you, grading your performance, and that's where the feedbacks came from. The observers had a better angle to see things clearly. But, I'm thinking, is it possible, in later training sessions, to video-tape the mock counseling sessions, give them a copy, allowing them to take a look at how they were doing by themselves…I think, they might feel better*.

Some participants suggested the trainees could expose the problems they encountered in real practice before the training started. In this way, mock sessions would fully reflect the real situations they face in their everyday work. After the training, there could be a second round of case scenarios to test if the participants had made progress.

### 3.2. Quantitative evaluation

The overall satisfaction over the training was confirmed by the structured evaluation questionnaire results. All participants agreed that the training was helpful to improve their consulting skills or techniques. On a 1–5 Likert scale with 1 being “strongly disagree” and 5 being “strongly agree,” the participants agreed that the training had a positive impact on their clinical practice (mean = 4.86), that they had a better awareness of their counseling style after the training (average score 4.45), and that they would recommend their colleagues to attend similar training program (mean = 4.84). They also agreed that the training helped them realize what they were good at (the average scores ranged from “flexible application of non-directive counseling” = 4.24 to “communication” = 4.52) and improve their counseling skills (the average scores ranged from “flexible application of non-directive counseling” = 4.60 to “the use of auxiliary means to explain professional terms” = 4.76). Moreover, the results from the survey confirmed the training helped participants develop more awareness about their practice and the learning progress needed. They realized they need to put more effort in all genetic counseling skills or techniques (the average scores ranged from “flexible application of non-directive counseling” and “the ability to gather information about the social background of the counselee” = 4.73 to “learning and updating their professional knowledge” = 4.90). All results further confirm what emerged earlier in the qualitative comments (see Table 2 for participants' assessment on the training course).

**Table 2:** Overall assessment on the training program.

	**No of respondents**	**Minimum score**	**Maximum score**	**Mean**
**1. Did this training help to improve your consulting skills?**				
1a. Explanation of terminology	49	3.00	5.00	4.65
1b. The use of auxiliary means to explain professional terms	49	4.00	5.00	4.76
1c. Flexible application of non-directive counseling	48	2.00	5.00	4.60
1d. The use of listening skills in consulting	49	2.00	5.00	4.65
1e. Ability to handle uncertainty	49	2.00	5.00	4.63
1f. Provision of follow-up supporting information (e.g., relevant sources, specialty referrals, patient group referrals)	49	2.00	5.00	4.61
**2. I realized that I need to put in more effort on a certain part**				
2a. Learning and updating of professional knowledge and skills	48	4.00	5.00	4.90
2b. Explanation of terminology	49	3.00	5.00	4.78
2c. Flexible application of non-directive counseling	49	3.00	5.00	4.73
2d. The ability to gather information about the social background of the counselee	49	3.00	5.00	4.73
2e. Ability to handle uncertainty	49	3.00	5.00	4.76
2f. Provision of follow-up supporting information (e.g., relevant sources, specialty referrals, patient group referrals)	48	3.00	5.00	4.79
**3. I realized that I was good at a certain part**				
3a. Listening	49	3.00	5.00	4.49
3b. Communication	48	3.00	5.00	4.52
3c. Flexible application of the principle of non-biased counseling	49	3.00	5.00	4.24
3d. Ability to gather background information	49	3.00	5.00	4.35
3e. Ability to handle uncertainty	49	1.00	5.00	4.37
3f. Provision of follow-up supporting information (e.g., relevant sources, specialty referrals, patient group referrals)	49	3.00	5.00	4.37
4. I have an in-depth understanding of my consulting pattern or style	49	3.00	5.00	4.45
**5. Overall evaluation of the training**				
5a. This training has had a positive effect on my clinical practice	50	4.00	5.00	4.86
5b. I will recommend my colleagues to attend a similar training	50	3.00	5.00	4.84
**6. Recommendations**				
6a. Increase expertise	50	3.00	5.00	4.84
6b. Increase time and opportunities for simulation	50	4.00	5.00	4.80
6c. Teach more counseling skills	49	3.00	5.00	4.78
6d. More diverse formats	50	3.00	5.00	4.80

## 4. Discussion

While genetic counseling has now been institutionalized as a profession in no less than 28 countries with 7,000 certified professionals worldwide (1), China is lagging, and its professionalization is still in the making. This study analyzed the first PEERS training experience conducted in Shanghai, China in 2016. The research confirmed the PEERS model to be a particularly effective tool for genetic counseling supervision (14); in fact it led the participants to deepen their counseling skills through exchange and reciprocal feedback and observations. The presence of an external observer combined with the possibility of reflexivity further enhanced their experiential learning. Self-awareness and self-reflection ultimately allowed participants to think over the needed skills and knowledge, together with the challenges inherent in genetic counseling.

Our findings have found that inadequate mastery of genetic and fetal medicine was regarded as a significant obstacle for most participants, which may result in perceived poor care by the patients; this reflects the overall lack of genetic training by genetic counselors in China (2). Further training is recommended to cope with the changing needs in the medical field; this is particularly true for China, where the effects of “three child policy,” combined with the introduction of next-generation sequencing (NGS) in prenatal diagnosis are creating a soaring demand for genetic counseling services (2). Besides, considering the uneven spread of medical resources of different regions across China, we suggest the training of knowledge and counseling skills could be separated into two parts. The training of knowledge can select and analyze the clinical cases that are more statistically frequent, but not yet common in regional hospitals. Due to the huge regional differences in China, trainees from different regions should be trained at different levels, including elementary, intermediate, and advanced classes, so that learning could be more targeted. For the training of communication skills, trainees could expose cases they consider more difficult or urgent, so as to make the training more relevant and reduce the gap between reality and fictionality.

Our analysis suggests that while genetic counseling should respect the autonomy, individuality, wellbeing, and freedom (2) of individuals and their families, participants realized the difficulties in providing information in a non-directive manner. Non-directive counseling has emerged as a challenge in previous research conducted in China (21); decades shaped by a “one child policy,” a discourse on a “quality population” and a society lacking a strong welfare system toward subjects with disabilities have resulted in a more directive counseling style by health professionals (21, 22). When providing genetic counseling services, these clinicians are bound by a duty to offer advice on reproductive issues for patients where the aim of genetic counseling in the Chinese context is to prevent genetic disorders. This is driven by China's socio-historico background—families in poverty are greatly burdened by children with genetic defects (4, 23). Further training is needed to promote more non-directive counseling practice, which may in turn enhance informed decision-making for patients and their families. Moreover, a lack of empathy and cultural sensitivity represented an obstacle to the full acknowledgment of the individuality of patients and their families. Similar to previous research (10), issues of trust toward medical professionals emerged as possible barriers to counseling practice; yet in this case it may be related to the difficulty of achieving trust during a mock session.

### 4.1. Strengths and limitations of the study

Overall, while the PEERS method was evaluated positively by the participants and mutual feedback were equally valued, efforts should be made to build an environment providing support and security to further establish a reciprocal peer relationship. Participants in fact lamented a feeling of insecurity and of being exposed under the public eye, which is not encouraging for discussion and exchange learning. For instance, the number of participants should be limited; 59 participants for training may not be an adequate number. Small-scale classes could instead facilitate communication, feedback, discussion, and study to enhance the learning experience.

## 5. Conclusion

The first of its kind in its application in China, our study has suggested that the PEERS model is beneficial toward the use in the practice of genetic medical education and its profession. Our participants were also satisfied with the training, and it was agreed that their consulting skills and techniques could be improved through this novel way of learning. Through our research, our findings have found the knowledge gaps needed to meet the demands of the changing needs in the medical system.

## Data availability statement

The raw data supporting the conclusions of this article will be made available by the authors, without undue reservation.

## Ethics statement

The studies involving human participants were reviewed and approved by Ethics Committee of the Shanghai First Maternity and Infant Hospital (No. KS1677). The patients/participants provided their written informed consent to participate in this study.

## Author contributions

DD and JZ conceptualized the assessment of the training course, acquired the funding needed for assessment, and developed the mixed-method study design. LS and SX prepared the contents and procedures of the training sessions, recruited participants, obtained the informed consent among the participants, and implemented the training course. BL, XC, and JZ conducted the ethnographic observation and interview, transcribed, and analyzed the qualitative data. DD analyzed the survey data. DD and SX drafted the original manuscript. EN, LZ, and FY extensively reviewed and edited the drafted original manuscript. All authors contributed to the article and approved the submitted version.
