# Supplementary material for: Applying and assessing the PEERS model on genetic counseling training in China: A mixed-method approach
Source: Front Med (Lausanne). 2023 Jan 11;9:986851. doi: 10.3389/fmed.2022.986851 (PMC9874665; doi:10.3389/fmed.2022.986851)
Supplement: Supplementary file 1 [file Table_1.pdf]

Supplementary Material

Supplementary Table 1: Demographic profiles of trainees

Province	City	Type of the hospital	Job title	Participant no.
**Jiangsu province**	Wuxi	Maternal and Children's Health	Chief Physician	1
			Associate Chief Physician	2
	Changzhou	Maternal and Children's Health	Associate Chief Physician	28
			Attending Physician	29; 30
	Suzhou	Maternal and Children's Health	Associate Chief Physician	46
			Department Head	47
	Huai'an	Maternal and Children's Health	Director of Obstetrics and Gynecology	48
**Guangdong province**	Shenzhen City	Maternal and Children's Health	Physician	3
			Associate Director	4
**Jiangxi province**	Jiujiang City	Maternal and Children's Health	Director	5; 6
	Nanchang City	Maternal and Children's Health	Associate Chief Physician	52
			Ultrasound Department	53; 55
			Associate Director of Obstetrics and Gynaecology	54
**Shandong province**	Linyi City	Maternal and Children's Health	Associate Director	7

	Jining City	Maternal and Children's Health	Director of Obstetrics and Gynaecology	18
**Zhejiang province**	Shaoxing City	Maternal and Children's Health	Associate Chief Physician	49; 50; 51
	Jiaxing	Maternal and Children's Health	Director of Obstetrics and Gynaecology	58
**Guangxi province**	Liuzhou	Maternal and Children's Health	Chief Physician	11
			Director of Obstetrics and Gynaecology	17
**Gansu province**	Lanzhou	Maternal and Children's Health	Prenatal Diagnostic Centre	19
			President	20
**Xinjiang province**	Ürümqi	Maternal and Children's Health	Obstetrics and Gynaecology	22
**Hebei province**	Shijiazhuang	Maternal and Children's Health	Associate Director	23; 25
			Obstetrician and Gynaecologist	24
**Liaoning province**	Dalian	Maternal and Children's Health	Department Head	26
			Associate Chief Physician	27
**Anhui province**	Hefei	Maternal and Children's Health	Director of Prenatal Diagnosis	31
			Associate Professor	32
**Fujian province**	Xiamen	Maternal and Children's Health	Director	34

	Fuzhou	Maternal and Children’s Health	Associate Chief Physician	45
**Hunan province**	Changsha	Maternal and Children’s Health	Associate Chief Physician	35
			Attending Physician	36
			Director	43
			Associate Chief Physician	44
**Guizhou province**	Guiyang	Maternal and Children’s Health	Associate Chief Physician	39
			Associate Director of Obstetrics and Gynaecology	40
			Attending Physician	41; 42
**Jilin province**	Changchun	Maternal and Children’s Health	President	56
			Department Head	57
**Shandong province**	Qingdao	Children’s Health	Associate Chief Physician	59
			Physician	60
**Zhejiang province**	Ningbo	Women and Children’s Health	Division Head	9
			Department Head	10
**Guangxi province**	Wuzhou	Maternal and Children’s Health	Director of Obstetrics and Gynaecology	12; 13
**Zhejiang province**	Ningbo	General, Teaching and Research Hospital	Director of Obstetrics and Gynaecology	8
			Ward Deputy Director	14
**Anhui province**	Hefei	Maternal and Children’s Health	Director of Obstetrics and Gynaecology	33
**Shanghai**			Chief Physician	37

			Attending Physician	38
**Jilin province**	Changchun	Maternal and Children’s Health	Prenatal Diagnostic Centre	61
**Qinghai province**	Xining	Non-profit organization	Chief Physician	15
